# Impact of social influence on users' continuance intention toward sports and fitness applications

**DOI:** 10.3389/fpubh.2022.1031520

**Published:** 2022-10-28

**Authors:** Zhiwen Li, Nian Du, Baojiao Wang, Clarissa Oteng-Darko

**Affiliations:** ^1^School of Management, Jiangsu University, Zhenjiang, China; ^2^School of Cultural Heritage and Information Management, Shanghai University, Shanghai, China

**Keywords:** social influence, continuance intention, sports and fitness application, perceived value, perceived pleasure

## Abstract

The purpose of this paper is to explore how social influence (SI), which is disaggregated into subjective norms (SN), social image (SIM), and social identity (SID), predicts perceived usefulness (PU), perceived pleasure (PP), and continuance intention (CI) toward sports and fitness applications. The underlying context is the socialization and gamification of exercise during the Covid-19 pandemic. Based on the theory of SI and the technology acceptance model, a theoretical framework was built where PU and PP mediate the influence of SI on CI, and proposed hypotheses were tested. The responses of 296 Keep users (a popular sports and fitness application in China) to a questionnaire survey were analyzed. SN and SIM were found to have significant positive effects on SID; SID has significant positive effects on PU and PP; both PU and PP have significant positive effects on the CI of users; SID and PU positively and significantly mediate the relationship between SN/SIM and CI; PU positively and significantly mediates the SID-CI relationship. However, the role of PP in mediating the influence of SI on CI is non-significant. This paper deepens the current understanding of the mechanisms that influence the relationship between SI and CI under the context of socialization and gamification services.

## Introduction

Today's lifestyle is becoming increasingly sedentary, which causes a rapid increase in the prevalence of ailments like cardiovascular problems, diabetes, and obesity. Most of these chronic diseases can be avoided by making lifestyle changes that include regular exercise and participation in sports. People typically have the tendency to focus on short-term rather than long-term rewards in their daily life (1). This often leads to the attempt to abandon physical exercise when short term results cannot be achieved from sports and fitness activities. As a result, individuals require sustained motivation to engage in physical activity.

Previous studies have shown that socialization services, particularly virtual communities (2) and gamification services (3–5), can encourage users' continued intention. Sports and fitness applications that offer gamification and socialization services are derived from this. They encourage users to participate in long-term activities by giving them short-term objectives, incentives, encouragement, and access to virtual communities. Virtual communities are also known as social networks that are interactive in nature and create a platform for users. The operation of websites has evolved in today's Web 2.0 era, and social media communities serve as the primary focus of development (6, 7). Businesses and other organizations use these virtual communities to market their brands, with associated fan sites becoming very prevalent. By providing engaging and copious content, as well as continued, uninterrupted interaction, the administrators of virtual communities try to draw more users and raise the market profile of the associated businesses.

Sports and fitness platforms are becoming increasingly homogenous, making it challenging for most users to assess the quality of such platforms. The role of social influence (SI) is becoming increasingly prominent. When predicting continuance intentions (CI), especially in the context of gamification, SI is vital (4), and the importance of the social aspects of sports gamification have been highlighted (6, 8). The number of available technological approaches that utilize SI and related psychological phenomena to steer human behavior toward sustainable, healthy, and otherwise beneficial behaviors is growing. However, research-based knowledge on whether these technological solutions with social features can actually motivate people to pick up and continue with encouraged behaviors is scarce.

The objective of this study is to explore the underlying process of the relationship between SI and users' CI toward sports and fitness applications. In this paper, several research gaps of previous studies on the role of SI in shaping users' CI are addressed. First, the interdisciplinary basis of SI across multiple research disciplines has led to the emergence of various conceptualizations with various labels and meanings (9). Researchers have coined a variety of terms to describe potential SI mechanisms associated with the adoption and continual use of technology. These include critical mass (10), perceived network externalities (11, 12), subjective norms [SN; (13)], group norms, and social identity [SID; (14, 15)], among others. These different terms reflect the conceptual complexity and heterogeneity of SI (16). As a result, most previous studies considered SI as a whole or lumped it under terms such as “social influence” or “social norm” to explain users' continuance behavior (4, 17). However, the underlying motivations, decision rules, and social processes differ both conceptually and theoretically (18). In this context, the concept of SI should be explained through a more clearly defined and appropriately tested subordination structure.

Second, previous studies focused on the relationship between SI and CIs through the mediating role of perceived value (17). How the role of different conceptualizations of SI work with perceived value to affect users' CI has not been explored so far. In the context of gamification and socialization services, this lack of knowledge calls for research that disaggregates SI to better understand how various conceptualizations of SI affect users' CI.

To fill this gap in the literature, first, SI was disaggregated into three different conceptualizations based on social influence theory (SIT). Particularly, SN are the primary conceptualization of SI. Several earlier studies conceptualized SI by viewing it as an identification process based on SN. To explain identification, two categories of conceptualization were employed by scholars: SID-related constructs [i.e., (19)] and social image (SIM)-related constructs [i.e., (6)]. Users can participate more actively in the virtual community and maintain active connections with other members because of the SID of the community (20, 21). Users are encouraged to continue participating in the virtual community because joining the virtual community improves their image and status (6). Therefore, in this study, SN, SID, and SIM are employed to represent SI.

Second, this paper examines how the conceptualization of SI works with perceived value to shape users' CI based on the technology acceptance model (TAM). Taking into account the function of sports and fitness application during the pandemic period, perceived value is represented by perceived usefulness (PU) and perceived pleasure (PP). They are incorporated into the theoretical framework to serve as the mediators in the relationship between the different conceptualizations of SI and users' CI.

This paper is organized into six sections, of which the Introduction is the first. Related theory, literature review, and research hypotheses presents the related theory, a succinct literature review, and hypotheses development. The methodology of the study is presented in Materials and methods. Results presents the results obtained. Discussion and conclusion provides a discussion and conclusions, and limitations and future research are provided in Section 6.

## Related theory, literature review, and research hypotheses

### Social Influence theory

The theory of how other people or organizations can affect people's behavior is explained by SIT. Venkatesh et al. (22) defined SI as “the degree to which an individual perceives that important others believe he or she should use the new system.” In recent years, SIT has been used to explore how people use information technology or systems, an approach that was first introduced by Davis (23). Venkatesh and Morris (24) examined gender, SIs, and how these factors affect people's acceptance and use of technology. Vanduhe et al. (4) combined task technology matching and social motivation to predict whether employees would accept gamification for training. Singh et al. (25) examined the impact of usage pressure, SI, and innovation effects analysis on the acceptance and recommendation of mobile wallet services.

Scholars have combined different theories to explore the relationship between SI and continued use of technology. However, the interdisciplinary basis of SI has led to various conceptualizations with different names and meanings, such as SN, group norms, SIM, and SID (22). These starkly different interpretations of SI pose a challenge for technology continuance usage research. It was very common for SN to be primarily conceptualized as SI (26). Many scholars have explored the relationship between SN and continuance use (27–29). In recent years, scholars began to conceptualize SI through SID and SIM (30, 31). In extension of this trend, this study conceptualizes SI through the three conceptualizations of SN, SID, and SIM.

SN refers to the perceived social pressure to perform or not to perform certain behaviors (32), mainly in view of what others expect. SID is the sense of belonging to and identification with a community and includes pride of being part of that community (33). Tajfel et al. (34) first proposed the theory of SID and defined “social identity as that part of an individual's self-concept which derives from his knowledge of his membership of a social group (or groups) together with the emotional significance attached to that membership.” In other words, SID refers to the individual's sense of identity relative to the group. Boulding (35) suggested that human image can convey a person's behavior, which reflects the subjective concept a person possesses. Moore and Benbasat (36) pointed out that image is the degree to which a person perceives that using innovation can improve their image or status. The concept of image can be applied to different fields, such as brand image, enterprise image, etc.

### Technology acceptance model

Based on the theory of reasoned action, Davis (23) developed the TAM. Davis indicated that the most crucial personal perceptions about using information technology were PU and perceived ease of use. PU is defined as “the degree to which a person believes that using a particular system would enhance his or her job performance.” Perceived ease of use is defined as “the degree to which a person believes that using a particular system would be free of effort.” In the information systems field, the TAM has been widely used to study the adoption of various technologies. Thereafter, Venkatesh and Davis (37) developed the TAM2 by adding SI and cognitive instrumental processes to predict the adoption of a certain information technology.

When a system is perceived as useful, persons tend to overcome the difficulty associated with its use to obtain the benefit its use promises (38). This suggests that PU may be a more important factor than perceived ease of use. Particularly, many studies highlighted the role of PU when analyzing users' CI toward health-related applications (17). In addition to PU, scholars suggested that PP is an equally important factor (17, 39, 40). Additionally, perceived enjoyment (i.e., pleasure) was integrated into the TAM and proven to be an important predictor of the continuance usage of health-related applications (41). Huang and Ren (42) recently suggested that people with low self-efficacy may place a higher value on entertainment features, such as gamification and social networking features. In summary, people with low self-efficacy may need an extra push to facilitate their self-regulation mechanisms to engage in physical activity regularly. More and more fitness applications are focusing on gamification and social applications, competition, achievements, and upgrades. In this context, this paper retains PU and PP to explain the users' CI.

### Hypotheses development

#### Subjective norms and social identity

Previous studies have demonstrated the connection between SID and SN. For instance, Terry et al. (43) found that the intention of people who strongly identify with a reference group to perform a certain behavior was influenced by perceived group norms. Thorbjørnsen et al. (44) suggested that SN positively influence SID. A customer will be more motivated to express particular ideas about the values and identity of in-group members if those beliefs are made more salient and/or if the drive to uphold those beliefs is strengthened. Reed (45) found that consumers are more likely to be influenced by SN when their SID is highly prominent. Therefore, the following hypothesis is proposed:

H1: *SN has a positive effect on SID*.

#### Social image and social identity

In addition to the expectations of significant others, the interactions with members of online sports and fitness communities also provide social benefits. Members' socialized participation is basically established in two ways: unilateral contributions as part of building one's reputation, and bilateral knowledge exchange for reciprocal behaviors (46). These rewards aid a person in identifying themselves as an in-group member or community opinion leader if the sharing of information and experiences increased their status and reputation. Helping others and exchanging information have such beneficial effects because of the sense of reciprocity associated with them (47). Previous studies have shown that SIM impacts SID (48). Users who believe that using sports and fitness applications will improve their status or image from the perspective of others also believe that this will result in favorable feedback from others about their behavior. Such favorable feedback further strengthens the users' identification with the community. Therefore, the following hypothesis is proposed:

H2: *SIM has a positive effect on SID*.

#### Social identity, perceived usefulness, and perceived pleasure

Song and Kim (3) first proposed that SID is a key determinant of the intention toward a particular technology or system in a virtual community service environment. Kwon and Wen (49) pointed out that SID significantly impacts user perceptions such as PU and perceived encouragement. Individuals with greater recognition in their communities will be more willing to participate and share information in online communities to continue using the system. Furthermore, Murray and Sabiston (50) suggested that SID is a predictor of PP because fostering SID with one's sport team may contribute to greater enjoyment of sports. In sports and fitness applications, individuals gain recognition from acceptance and compliance to SIs, thus gaining emotional experiences such as PP (51). Therefore, the following hypotheses are proposed:

H3: *SID has a positive effect on PU*.

H4: *SID has a positive effect on PP*.

#### Perceived usefulness, perceived pleasure, and continuance intention

Motivational theory is frequently used to explain how people behave when accepting information technology. Davis et al. (52) found that both extrinsic (usefulness) and intrinsic (pleasure) factors influence the motivation to use systems. In addition, many scholars have found that both intrinsic motivation (pleasure) and extrinsic motivation (usefulness) affect an individual's intention to use information technology (53, 54). If an individual perceives a system as useful, this individual will actively use it. Research confirmed that users' PU has a positive effect on the use of information technology (55–58).

PP is an important factor in motivating users to use new technologies (52, 59, 60), especially hedonic systems (52, 54, 61, 62), where individuals engage in a certain activity for pleasure (62). In fact, PP is part of all performance consequences of technology use, defined as the extent to which the use of technology itself is considered pleasurable (61–64). Users are more likely to repeatedly utilize an application when they are satisfied with it (39). Therefore, the following hypotheses are proposed:

H5: *PU has a positive effect on CI*.

H6: *PP has a positive effect on CI*.

#### Social identity and continuance intention

SID helps users to actively interact and participate with other members of shared social network groups (14, 20, 65). Song and Kim (3) identified SID as a key factor influencing the intention to use a particular technology or system in online activities. More specifically, Vanduhe et al. (4) found that PU is a significant mediator of the effects of social recognition and SI on CI. If individuals had higher recognition of their communities, they will have greater willingness to participate and share information in online communities (48). Perceived usefulness has the potential to mediate the effect of social identity on persistent intentions. Murray and Sabiston (50) found that enjoyment (pleasure) mediates the relationship between SID and the cessation of sports activities. This is similar to the context of continuous exercise and fitness application use, where PP may play a mediating role. Therefore, the following hypotheses are proposed:

H7: *The effect of SID on CI is mediated by PU*.

H8: *The effect of SID on CI is mediated by PP*.

#### Subjective norms and continuance intention

The personal cognitive behavior of consumers is affected by the virtual communities and peers they engage with (66). Consumers will be persuaded to join the community and use the sports and fitness system by their peers if they anticipate that their peers will also use the platform. According to the theory of rational action, the behavioral intentions of individuals are influenced by SN (65). Furthermore, SN has implications for individuals' intention to use information technology (67, 68). In addition, SN is also assumed to be an important determinant of the CI to use certain systems. Studies have shown the effect of SN on the prolonged use of instant messaging (14). To lower the predicted risk, users are more mindful about other people's conduct or opinions in a network setting that contains numerous unknown aspects. As a result, SN has an even stronger impact.

However, this effect is not apparent. Reed (45) found that consumers are more likely to be influenced by SN when their SID is highly prominent. SID mediates the effect of SN on intention (69, 70). Chen et al. (71) found that social identity plays an essential intermediary role in the participation mechanism. Moreover, perceived value is a significant mediator of the effects of SI on CI (4). For example, Vanduhe et al. (4) found that perceived usefulness is a significant mediator of the effects of reputation, social recognition and social influence on continuance intention. This means that the influence of SN on intention is mediated by SID and PU/PP. Therefore, the following hypotheses are proposed:

H9: *The effect of SN on CI is mediated by SID and PU*.

H10: *The effect of SN on CI is mediated by SID and PP*.

#### Social image and continuance intention

The desire to increase one's social status is a significant cause for people employing innovative technologies (72, 73). People adopt innovative technologies and build social differences by developing their SIM (74, 75). Therefore, one of the potential benefits of using sports and fitness applications is gaining positive SIM. Previous studies have uncovered a positive correlation between SIM and CI (6, 76). Only when users believe that using a certain system will improve their status and image, they will participate more actively (6).

SI impacts users' CIs through perceived value (77). When users get more attention and positive feedback from the community, they will slowly accumulate fans, which will undoubtedly enhance the users' status in the community as these communities are driven by their fan/follower bases. When users have a large number of fans, they feel a stronger sense of belonging to the community, and the joy they perceive from the large number of fans prompts users to continue using the system and become involved more actively. Therefore, the following hypotheses are proposed:

H11: *The effect of SIM on CI is mediated by SID and PU*.

H12: *The effect of SIM on CI is mediated by SID and PP*.

**Figure 1:**
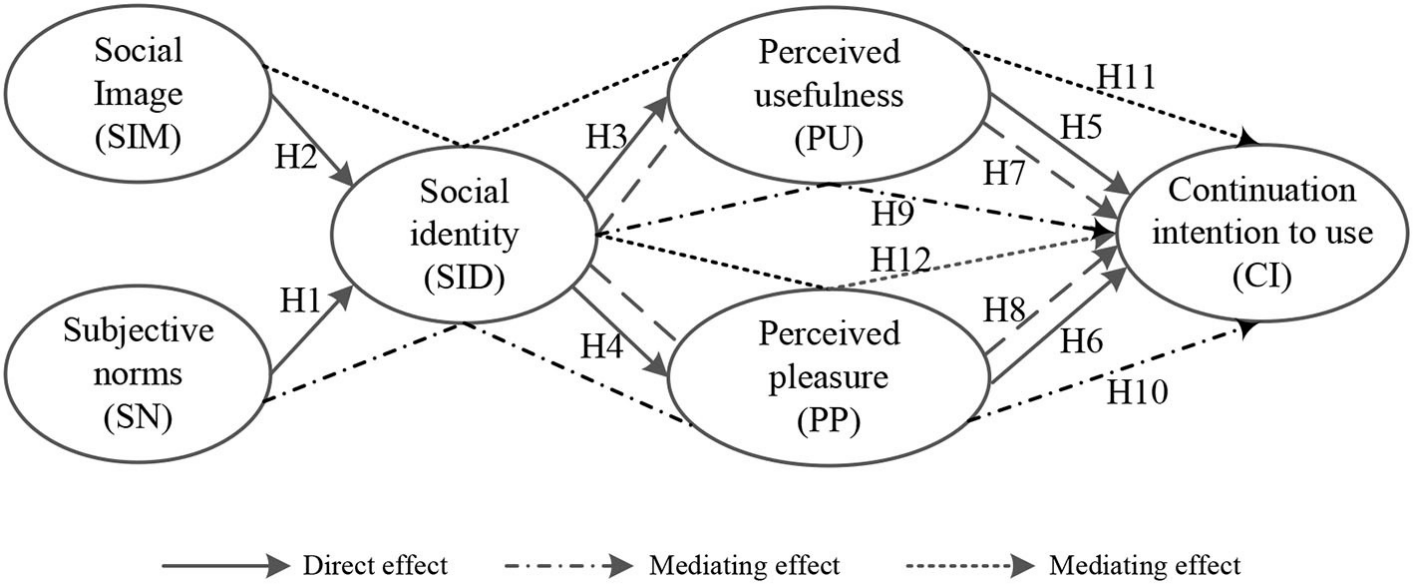
The theoretical framework.

Based on the above analysis, the theoretical framework of this study is presented in Figure 1.

## Materials and methods

### Data collection

In this study, users of the sports and fitness community of Keep, a popular sports and fitness application in China, were investigated. Keep was launched on February 4, 2015. According to China Insights Consultancy, Keep had the largest online fitness user base in China, with a monthly average of 34.4 million active users in 2021. The introduction of social interaction functions in the Keep community is a powerful supplement to the core fitness experience provided by the platform. The active community brings stronger incentives, a more intense sense of competition, and closer mutual connection, which helps to enhance user loyalty. In 2021, the Keep community had a total of 1.7 billion interactions (including posts, likes, and comments). This identifies the Keep community as a sport and fitness system that provides gamification and socialization services. Therefore, it is an ideal research target for this study.

A web-based questionnaire was designed on “Wenjuanxing,” which is a very popular online survey platform. The link to the questionnaire was posted in the Keep community from 17th February 2022 to 10th March 2022 and participants were invited to provide their responses to the questionnaire. The questionnaire was online for 4 weeks and a total of 417 responses were collected. Of these, 77 were excluded because of unfinished answer, and 44 were excluded because of missing values. The number of valid responses was 296, resulting in a 70.98% valid response rate, which meets the requirements of the number of samples for structural equation modeling (SEM) analysis. The results of the demographic analysis are detailed in Table 1.

**Table 1:** Description of the respondents (*N* = 296).

	**Characteristics of respondent**	**Frequency (*n*)**	**Percentage (%)**
Gender	Male	145	49.0
	Female	151	51.0
Age	20 years old and below	56	18.9
	21–30years old	142	48.0
	31–40 years old	70	23.6
	41–50 years old	21	7.1
	51 years old and above	7	2.4
Education background	Junior high school and below	8	2.7
	High school or secondary school	68	23.0
	College or undergraduate	148	50.0
	Master's degree or above student	72	24.3
Monthly income	800 ¥ and below	17	5.7
	801–2000 ¥	110	37.2
	2001–4000 ¥	72	24.3
	4001–6000 ¥	53	17.9
	6001–8000 ¥	33	11.1
	More than 8001 ¥	11	3.7
Region	Northeast China	5	1.7
	East China	96	32.4
	North China	20	6.8
	Central China	57	19.3
	South China	48	16.2
	Southwest China	54	18.2
	Northwest China	16	5.4

### Measures

A 5-point Likert scale was recommended by scholars in the scale development process because it provides an easier response process for respondents but does not pose a major disadvantage compared to the 7-point Likert scale in terms of reliability (78). The applied Likert scale ranges from strongly disagree to strongly agree, coded as 1 to 5, respectively.

Specifically, the six constructs of this study consist of 19 sub-variables. The items originate from previous research and are modified according to the research topic (as shown in Table 2). The questionnaire also collects demographic data of respondents, such as age, gender, educational background, monthly income and region.

**Table 2:** Measurement instruments.

**Construct**	**Name**	**Included/ Total items**	**Adapted from**
SN	Subjective norms	3/4	Venkatesh and Davis, (37) Rhodes and Courneya, (79) Peng et al., (80)
SIM	Social image	4/4	Venkatesh and Davis, (37); Moore and Benbasat, (36)
SID	Social identity	3/3	Zhou and Li, (14)
PU	Perceived usefulness	3/3	Venkatesh and Davis, (37) Bhattacherjee et al., (81)
PP	Perceived pleasure	3/3	Moon and Kim, (53)
CI	Continuance intention	3/3	Bhattacherjee et al., (81) Moon and Kim, (53)

The scales of CI and PU were adapted from Venkatesh and Davis (37), Bhattacherjee et al. (81), and Moon and Kim (53) who focused on the adoption and continual use of information technology. The scale of SIM was adapted from Venkatesh and Davis (37) as well as Moore and Benbasat (36), because both studies considered the improvement of the image resulting from the adoption of the innovation. The scale of SN was adapted from Venkatesh and Davis (37), Rhodes and Courneya (79, 80) because they addressed the relationship between SN and behavior. The scale of PP was adapted from Moon and Kim (53), who introduced pleasure as a factor (intrinsic motivation), reflecting users' intrinsic beliefs in world-wide-web acceptance. The scale of SID was adapted from Zhou and Li (14), who studied continuance use from the perspective of SI.

To critically evaluate the influence of SI on users' CI toward Keep, the following outcomes of SEM-based statistical tests help to either justify or nullify the hypotheses proposed in this paper. Firstly, prior to any statistical analysis, all negative statements were recoded. Secondly, a factor analysis was performed on all variables. For the characteristics of SN, three factors were produced out of four. For characteristics of SID, three factors were produced out of three. For the characteristics of SIM, four factors were produced out of four. For the characteristics of PU, three factors were produced out of three. For the characteristics of PP, three factors were produced out of three. For the characteristics of CI, three factors were produced out of three. Based on the statements of each factor, the factors were then labeled.

### Measurement model

SEM was used for data analysis through the covariance-based SEM (CBSEM) software AMOS version 21. CBSEM clearly outperforms partial least squares in terms of parameter consistency making it preferable in terms of parameter accuracy if the sample size exceeds a certain threshold (i.e., 250 observations) (82).

Data analysis was conducted in the following three steps: (1) data normality test. (2) reliability and construct validity test, and (3) structural model analysis. To assess whether the measure of CI toward sports and fitness applications includes six constructs, data were tested for normality and confirmatory factor analysis (CFA) was performed to examine the reliability and validity of the measurement model for the research variables (83, 84). The comparative fit index (CFI), a Tucker-Lewis index (TLI) > 0.90, and a root-mean-square error of approximation (RMSEA) **≤** 0.06 were used as criteria for evaluating goodness of-fit indices (85). Additionally, a SEM analysis was performed by building a mediation model to test the structural model. Finally, to assess the mediation hypothesis, a Bootstrap procedure was performed using the 95% bias corrected confidence interval to assess the mean indirect mediation.

## Results

### Descriptive statistics

Table 3 shows the result of descriptive statistics for all variables. The mean value of SN was 3.33–3.49, and the mean of SID was 3.60–3.64. The mean value of SIM was 3.15–3.48, and the mean value of PU was 3.85–3.92. The mean value of PP was 3.54–3.85, and the mean value of CI was 3.72–3.97. The skewness and kurtosis for all constructs in the study show an adequate range of ±2, which agrees with the symmetry of the sample distribution (86).

**Table 3:** Descriptive analysis.

		**N**	**Min**	**Max**	**Mean**		**SD**	**Skewness**		**Kurtosis**	
		**Stat**	**Stat**	**Stat**	**Stat**	**Std. Error**	**Stat**	**Stat**	**Std. Error**	**Stat**	**Std. Error**
SN	SN1	296	1	5	3.49	0.058	0.991	−0.287	0.142	−0.442	0.282
	SN2	296	1	5	3.36	0.067	1.154	−0.454	0.142	−0.634	0.282
	SN3	296	1	5	3.33	0.069	1.179	−0.299	0.142	−0.716	0.282
SID	SID1	296	1	5	3.60	0.056	0.958	−0.534	0.142	−0.048	0.282
	SID2	296	1	5	3.64	0.059	1.022	−0.600	0.142	−0.088	0.282
	SID3	296	1	5	3.61	0.054	0.921	−0.630	0.142	0.256	0.282
SIM	SIM1	296	1	5	3.30	0.068	1.162	−0.404	0.142	−0.628	0.282
	SIM2	296	1	5	3.38	0.060	1.031	−0.417	0.142	−0.437	0.282
	SIM3	296	1	5	3.15	0.071	1.225	−0.158	0.142	−0.859	0.282
	SIM4	296	1	5	3.48	0.061	1.044	−0.584	0.142	−0.115	0.282
PU	PU1	296	1	5	3.85	0.049	0.836	−0.794	0.142	0.834	0.282
	PU2	296	1	5	3.92	0.053	0.906	−0.611	0.142	0.049	0.282
	PU3	296	1	5	3.92	0.048	0.833	−1.078	0.142	1.992	0.282
PP	PP1	296	1	5	3.54	0.059	1.014	−0.533	0.142	−0.230	0.282
	PP2	296	1	5	3.56	0.060	1.027	−0.619	0.142	−0.104	0.282
	PP3	296	1	5	3.85	0.050	0.856	−0.388	0.142	−0.289	0.282
CI	CI1	296	1	5	3.72	0.055	0.953	−0.610	0.142	0.170	0.282
	CI2	296	1	5	3.78	0.054	0.928	−0.607	0.142	0.162	0.282
	CI3	296	1	5	3.97	0.051	0.881	−0.720	0.142	0.251	0.282

### Structural model

#### Confirmatory factor analysis of variables

Six constructs (i.e., SN, SIM, SID, PU, PP, and CI) were used as the main constructs. CFA was used to test the compatibility of the study for reliability and validity analyses. In addition, CFA helps to understand whether the measurements of constructs are consistent (83).

Three criteria were used to evaluate both the reliability and validity of CFA (87, 88). (1) The reliability of each index is evaluated *via* standardized factor loadings (factor loading > 0.5). (2) Cronbach's α and composite reliability are used to measure reliability (composite reliability > 0.6 and Cronbach's α > 0.7). (3) the average variance extracted (AVE) is used to measure convergent validity (AVE higher than 0.5). As shown in Table 4, the factor loadings of all variables range between 0.6 and 0.9, and factor loading is significant. Cronbach's α is > 0.8, composite reliability ranges between 0.7 and 0.9, and AVE ranges between 0.4 and 0.6. Although the AVE for PU is 0.466, it is still within the acceptable range. This result indicates that these empirical data reach convergent validity.

**Table 4:** CFA analysis results.

		**Ustd**	**S.E**.	***t*–value**	**P**	**Std**	**SMC**	**C.R**.	**AVE**	**Cronbach's alpha**
SN	SN3	1.000				0.648	0.420	0.803	0.512	0.816
	SN2	1.336	0.155	8.623	***	0.885	0.783			
	SN1	0.840	0.092	9.126	***	0.647	0.419			
SID	SIM1	1.000				0.760	0.578	0.822	0.536	0.820
	SIM2	0.904	0.073	12.322	***	0.775	0.601			
	SIM3	1.122	0.088	12.820	***	0.810	0.656			
	SIM4	0.848	0.071	11.913	***	0.718	0.516			
SIM	SID1	1.000				0.726	0.527	0.797	0.569	0.851
	SID2	1.222	0.118	10.338	***	0.831	0.691			
	SID3	0.926	0.091	10.227	***	0.699	0.489			
PU	PP1	1.000				0.788	0.621	0.813	0.466	0.810
	PP2	0.827	0.108	7.673	***	0.644	0.415			
	PP3	0.681	0.089	7.656	***	0.636	0.404			
PP	PU1	1.000				0.678	0.460	0.808	0.513	0.806
	PU2	1.007	0.125	8.049	***	0.630	0.397			
	PU3	1.132	0.143	7.921	***	0.770	0.593			
CI	CI1	1.000				0.685	0.469	0.827	0.545	0.825
	CI2	1.160	0.120	9.698	***	0.816	0.666			
	CI3	0.974	0.100	9.785	***	0.722	0.521			

*p < 0.05; **p < 0.01; ***p < 0.001.

Discriminant validity analysis means that, when multiple indicators of a trait show a certain degree of convergence, the indicators of the trait should be negatively correlated with the measure of its opposing trait. In other words, discriminant validity is mainly used to test the degree to which measures of different traits are unrelated. In this study, the correlation coefficient between traits is tested by the bootstrap method, and the confidence interval is 95%. If the confidence interval of each trait does not contain 1, there is discriminant validity (89). According to the analysis result of this study (shown in Table 5), the confidence intervals of each trait do not contain the correlation coefficient 1 at a 95% confidence level. This implies that there is good discrimination among traits.

**Table 5:** Discriminant validity analysis results.

**Relationship**			**Estimate**	**Bias–corrected**		**Percentile**	
				**Lower**	**Upper**	**Lower**	**Upper**
SN	< —>	SIM	0.769	0.672	0.861	0.669	0.859
SN	< —>	SID	0.673	0.559	0.768	0.565	0.773
SN	< —>	PP	0.655	0.517	0.778	0.518	0.778
SN	< —>	PU	0.542	0.395	0.665	0.393	0.665
SN	< —>	CI	0.665	0.548	0.773	0.550	0.774
SIM	< —>	SID	0.689	0.566	0.794	0.566	0.794
SIM	< —>	PP	0.630	0.504	0.741	0.504	0.741
SIM	< —>	PU	0.453	0.312	0.595	0.304	0.588
SIM	< —>	CI	0.607	0.485	0.714	0.483	0.712
SID	< —>	PP	0.674	0.532	0.794	0.534	0.796
SID	< —>	PU	0.564	0.412	0.703	0.410	0.699
SID	< —>	CI	0.651	0.517	0.764	0.520	0.767
PP	< —>	PU	0.733	0.575	0.862	0.576	0.863
PP	< —>	CI	0.767	0.657	0.863	0.661	0.866
PU	< —>	CI	0.700	0.579	0.807	0.579	0.807

In the theoretical model, PU/PP mediates the SID–CI link, and SID and PU/PP mediate both the SN–CI link and the SIM–CI link. The statistical results based on model fit indices show that all values of CFA indicators are within their threshold of excellence.

The observed value of χ^2^/DF is 1.722 (which is < 3). The DF (degree of freedom) is the reference value for determining whether the value is too large, and the RMSEA value is 0.050 (< 0.08). The GFI (Goodness of Fit Index) value is 0.918 (> 0.90). The χ2/DF, RMSEA and GFI are the proportion of the sample covariance matrix explained by the model covariance matrix. The higher the value is, the better the model fit tends to be.

The TLI value is 0.948 (> 0.90), the CFI value is 0.956 (> 0.90), the NFI (Normed-Fit Index) value is 0.902 (> 0.90), TLI, CFI and NFI are the extent to which the fit of the study model has improved in comparison to the statistical base model. The higher the value is, the better the model fit tends to be. These results indicate that the proposed measurement model in this research fits the empirical data well.

#### Test of hypotheses

Path analysis was employed to test the hypotheses. Table 6 shows the results of hypothesis testing, including their standardized regression weight (Std), unstandardized regression weight (Ustd), standard error (S.E.), *t*-value, and *p*-value. According to the findings of this study, the path coefficients provide empirical support for the first six hypotheses tested in this study because this technique can be used to test multiple levels of a theoretical framework.

**Table 6:** Path coefficients and hypothesis tests.

**Hypotheses**	**Relationship**	**Std**	**Ustd**	**S.E**.	***t*–value**	**p**	**Result**
H1	SN → SID	0.448	0.370	0.091	4.077	***	Supported
H2	SIM → SID	0.396	0.372	0.101	3.686	***	Supported
H3	SID → PU	0.673	0.626	0.075	8.298	***	Supported
H4	SID → PP	0.811	0.830	0.091	9.105	***	Supported
H5	PU → CI	0.365	0.377	0.097	3.908	***	Supported
H6	PP → CI	0.568	0.534	0.099	5.372	***	Supported

*p < 0.05; **p < 0.01; ***p < 0.001.

Analyses showed that SN has a positive impact on SID (Std = 0.448, S.E. = 0.091, *t*-value = 4.077 > 2, *p*-value = 0.000 < 0.01). In other words, SN plays a critical role in SID, which is consistent with previous studies, such as Thorbjørnsen et al. (44). Therefore, H1 was supported.

The test of H2 showed that the Std is 0.396, the S.E. is 0.101, the *t*-value = 3.686 > 2, and the *p*-value = 0.000 < 0.01. Therefore, H2 was supported. This finding is consistent with a recent study which argued that SIM had a positive impact on SID if it was used effectively (48).

According to the analysis, SID has a positive impact on the PU of Keep users, which supports H3. The results showed that the Std is 0.673, the S.E. is 0.075, the *t*-value = 8.298 > 2, and the *p*-value = 0.000 < 0.01. Previous research found similar results, indicating that SID significantly affects PU (49).

H4 is also supported because the Std is 0.811, the S.E. is 0.091, the *t*-value = 9.105 > 2, and the *p*-value = 0.000 < 0.01. Regarding H5, the results of the study showed that the Std is 0.365, the S.E. is 0.097, the *t*-value = 3.908 > 2, and the *p*-value = 0.000 < 0.01. Therefore, H5 was supported. These findings are consistent with the results of previous studies, demonstrating that PU significantly influences users' intention to use technology [i.e., (58)].

The results showed a significant impact of PP on CI (Std = 0.568, S.E. = 0.099, *t*-value = 5.372 > 2, and *p*-value = 0.000 < 0.01). Therefore, H6 was supported, and the result echoes previous studies examining the effect of PP on CI [i.e., (39)].

**Figure 2:**
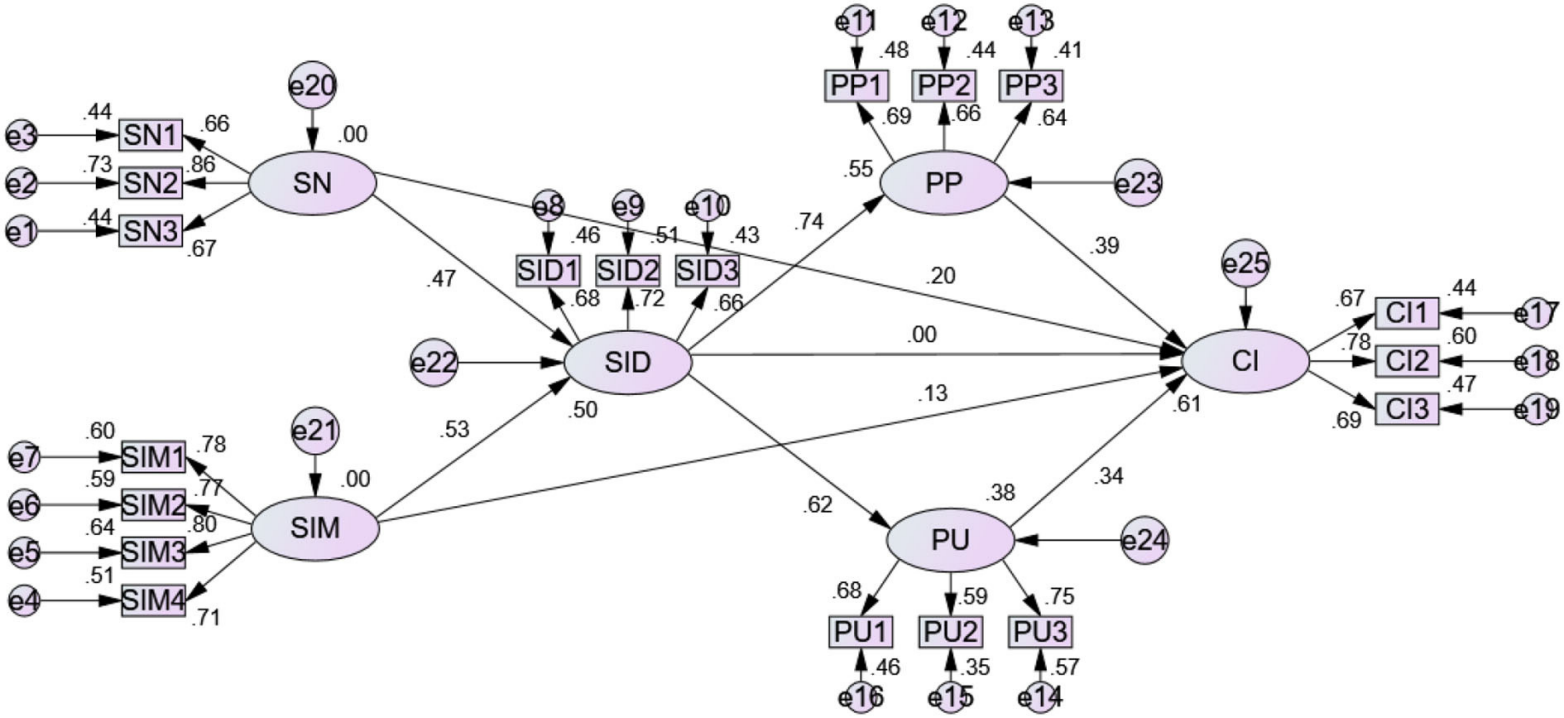
Mediation model.

The bootstrap method was used to analyze mediating effects. Compared to traditional methods, the advantage of the bootstrap method is that it can directly test the indirect effects of independent variables on the dependent variables, while not requiring the mediating effects to follow a normal distribution (90). Furthermore, the bootstrap method generates more reliable results with smaller samples (91). A sample size of 5,000 was selected, and percentile and bias-corrected bootstrap was selected as confidence interval method. The mediation model is acceptable (as shown in Figure 2, CMIN/DF = 2.700, IFI = 0.899, GFI = 0.891, CFI = 0.898, NFI = 0.849, and RMSEA = 0.076).

Table 7 presents the results of mediating analysis using AMOS 21.0 (CBSEM). If the indirect effects are not statistically significant, it was concluded that no mediating effect exists (92). The mediating effect works if the indirect effect is significant and the confidence interval does not contain zero and z (point estimation/SE_in_) ≥ 1.96 (93).

**Table 7:** Mediating effect test results.

**Estimated**	**Point estimation**	**Product of coef**.		**Bias–corrected**		**Percentile**		**Result**
		**SE**	**Z**	**Lower**	**Upper**	**Lower**	**Upper**	
H7:SID–PU–CI	0.279	0.101	2.762	0.123	0.541	0.108	0.509	Supported
H8:SID–PP–CI	0.284	0.149	1.906	0.074	0.641	0.072	0.638	Not supported
H9:SN–SID–PU–CI	0.077	0.032	2.406	0.032	0.164	0.026	0.154	Supported
H10:SN–SID–PP–CI	0.104	0.058	1.793	0.029	0.253	0.026	0.244	Not supported
H11:SIM–SID–PU–CI	0.092	0.037	2.486	0.040	0.201	0.031	0.177	Supported
H12:SIM–SID–PP–CI	0.125	0.070	1.786	0.032	0.308	0.030	0.298	Not supported

PU positively and significantly mediates the SID-CI link (z = 2.762 > 1.96, bias-corrected CI is [0.123, 0.541], percentile CI is [0.108, 0.509]). SID and PU positively and significantly mediate the SN-CI link and the SIM-CI link (z = 2.406 > 1.96, bias-corrected CI is [0.032, 0.164], percentile CI is [0.026, 0.154]; z = 2.486 > 1.96, bias-corrected CI is [0.040, 0.201], percentile CI is [0.031,0.177]). As a result, H7, H9, and H11 are all supported. This is consistent with previous studies reporting that PU mediates the relationships among social recognition and SI on CIs [i.e., (4)].

However, the mediating effect of PP on the relationship between SID and CI is not significant because z = 1.906 < 1.96; therefore, H8 is not supported. The mediating effect of SID and PP on the relationship between SN and CI is also not significant, because z = 1.793 < 1.96. Likewise, the mediating effect of SID and PP on the relationship between SIM and CI is not significant because z = 1.786 < 1.96. As a result, H10 and H12 are also not supported. The results suggest that Keep's in-app gamification services are not well developed, and using Keep produces insufficient pleasure for users, which limits the mediating role of PP.

## Discussion and conclusion

In today's era of the mobile Internet, how to retain users has become the priority for mobile applications. What many mobile applications, such as Keep, have in common is their attempt to engage people to maintain their ongoing behavior through the SI of virtual communities (27). The role of SI on the users' CI has been highlighted by both scholars and practitioners. This study examined how SI (mediated by perceived value) affects CI toward the users of sports and fitness applications by disaggregating the SI into SN, SIM, and SID.

Using SEM to analyze the proposed model, this paper provides empirical evidence that the SI of virtual communities positively impacts users' CI toward sports and fitness applications during the COVID-19 pandemic. Based on the survey results, SN and SIM can help the users of sports and fitness applications to improve their SID to the community, and then recognize, participate in, and perform their activities. Specifically, SID and PU serve as mediating factors in the relationships either between SN and CI or between SIM and CI. Furthermore, PU serves as the mediating factor in the relationship between SID and CI, but PP neither mediates the SN-CI link, the SIM-CI link, nor the SID-CI link. This result suggests that SI affects users' CI only by affecting their PU. The most important connection point in the SI process (i.e., SID) will increase users' perception of the usefulness of the functions of sports and fitness applications, and this perception will greatly and positively affect the users' continued intention to use the application.

### Theoretical implications

This study has the potential to contribute to the field of CI research in the following three aspects:

First, when examining its role, previously, SI was mostly considered as a whole under the term “social influence” or “social norm” (4, 17). Few studies have attempted to disaggregate it and further explore its role through different conceptualizations. The present study disaggregated SI into SN, SIM, and SID and examined the impact of different conceptualizations on users' CI. Different from previous studies, this study sheds light on the conceptualizations and influencing mechanism of SI.

Second, the mediating effect of either SID or PU on users' CI has been highlighted previously (4, 69, 70). However, their chain mediating effect has been under-explored. In this study, both SID and PU were integrated to explore their chain mediating effect on users' CI. Therefore, the underlying process of the relationship between SI and users' CI was clarified.

Third, previous studies showed that it is not easy for users to maintain healthy behaviors over time (94). In addition to socialization, gamification has been regarded as an efficient way to motivate healthy behaviors is users. However, few scholars have examined the mediating role of PP in shaping users' CI. In this study, not only the mediating effect of PP on users' CI was explored, but also the chain mediating effect of both SID and PP on users' CI.

### Practical implications

Sports and fitness professionals can benefit from the current study in the real-world in significant ways. First, sports and fitness operators should be dedicated to satisfying users' expectations for real-time interactive communication. This can be achieved by creating virtual communities and working with social media, as doing so will encourage users to exchange messages with others. Real-time interactive communication makes communication with other users or online sports and fitness providers easy. Consequently, users can get the information they need during the interaction or become friends with users with shared interests. In addition, sports and fitness suppliers should provide users with information on how to improve the efficiency of sports and fitness, useful sports and fitness equipment, and sports and fitness health knowledge in the sports and fitness community in a timely manner. Such information enables users to gain knowledge effectively and make quick decisions. Through long-term content marketing that reflects interests, current events, or hot topics, interest and active participation in discussions and interactions can be generated in users. This enhances users' sense of belonging to the sports and fitness community and their intention and behavior to continue using the application.

Second, the influence of key opinion leaders in the sports and fitness industry can have a positive publicity role. Most sports and fitness users, according to the survey, are between the ages of 21 and 30. This age group is active and passionate, and they frequently share their usage of sports and fitness platforms and services through social media and other channels. Moreover, people in this age group are more willing to accept the use of new technologies and new products, as doing so can enhance the status of the individual. Therefore, operators should cooperate with key opinion leaders who are in line with the brand's image. Users are less inclined to abandon a platform or group if the group contains many people they are familiar with. However, when an issue arises within the platform or community that the user cannot overlook or accept, users not only leave but also encourage those around them to do the same. Therefore, when sports and fitness platforms develop according to the network model, it is necessary to pay attention to relationship management, while improving the service quality of the platform and the ability to deal with unexpected problems. Doing so can avoid the occurrence of “bad news travels fast, while good news take the scenic route.”

Finally, although the SI of virtual communities will enable users to discover the value of the platform, sports and fitness operators should consider whether their platform is more useful than other existing sports and fitness platforms. Improving the quality of sports and fitness courses, knowledge, equipment, data monitoring, paying attention to the setting of gamification mechanisms in the system, and providing users with high-quality, personalized, and gamified sports and fitness services can help the sports and fitness platform to operate continuously and be loved by its users. This will arouse the intention of users to recommend the platform to those around them. Perceived pleasure is supposed to influence users' continuance intention. However, the non-significant mediating role of perceived pleasure showed by this study suggests that at this stage, social influence does not have a significant impact on the continuance intention of Keep users through perceived pleasure. This urges Keep to increase the number or improve the quality of in-app gamification services to stimulate users' pleasure in order to increase their continuance intention.

## Limitations and future research

In the following, research limitations are summarized to favorably contribute to future studies. First, this study was based on a sample of Keep users in China. However, as culture or preferences of each country and region differ, whether the results of this study can be generalized to others countries is an interesting question for future work.

Second, this study presents a cross-sectional measurement of sample collection at a certain point in time. Therefore, the developed theoretical model cannot explain the factors of users' CI over a specific period or the changes in user intention over time. The authors recommend that scholars make time series observations of homogeneous samples to assess the transition from initial use to sustainable use in depth.

Third, this study explores individual and community-related impacts from a community-impact perspective, but it does not discuss other environments outside the community or the relationship between sports and fitness operators and users. Future research can explore the impact between sports and fitness operators, as well as platforms and users.

Finally, this study mainly explored the characteristics and environment of users and sports and fitness apps in the sports and fitness community without having a particularly deep understanding of the influencing mechanisms of each part. For example, in terms of platform, the impact of the interface design, process, and stability of the sports and fitness application on the CI of sports and fitness users can be further explored. This can achieve a deeper and more thorough understanding of the impact of the continuous use of sports and fitness applications.

## Data availability statement

The raw data supporting the conclusions of this article will be made available by the authors, without undue reservation.

## Author contributions

Conceptualization and software were contributed by ZL. Data curation and formal analysis were contributed by ND and BW. Writing—original draft was contributed by ND and ZL. Revised manuscript: ZL, ND, and CO-D. All authors contributed to the paper and approved the submitted version.

## Conflict of interest

The authors declare that the research was conducted in the absence of any commercial or financial relationships that could be construed as a potential conflict of interest.

## Publisher's note

All claims expressed in this article are solely those of the authors and do not necessarily represent those of their affiliated organizations, or those of the publisher, the editors and the reviewers. Any product that may be evaluated in this article, or claim that may be made by its manufacturer, is not guaranteed or endorsed by the publisher.

## Appendix A: Research items.

**Table:**

SN	People who are important to me think that I should use Keep.
	People who influence my behavior think that I should use Keep.
	People I look up to expect me to use Keep.
SID	I have a strong feeling of belonging toward Keep.
	I am an important member of Keep.
	My self–image overlaps with the identity of Keep.
SIM	People in my exercise group who use Keep have more prestige than those who do not.
	People in my exercise group who use Keep have a high profile.
	Having Keep is a status symbol in my exercise group.
	Using Keep improves my image within the exercise group.
PU	Using Keep enhances my effectiveness in my exercise.
	Using Keep in my exercise increases my productivity.
	I find Keep to be useful in my exercise.
PP	When interacting with Keep, I do not realize the time elapsed.
	When interacting with Keep, I am not aware of any noise
	Using Keep keeps me happy for my exercise.
CI	I intend to continue using the Keep on my exercise.
	I will frequently use Keep in the future.
	I will strongly recommend others to use Keep.
